# A Smart Diaper System Using Bluetooth and Smartphones to Automatically Detect Urination and Volume of Voiding: Prospective Observational Pilot Study in an Acute Care Hospital

**DOI:** 10.2196/29979

**Published:** 2021-07-30

**Authors:** Jae Ho Cho, Jung-Yeon Choi, Nak-Hyun Kim, Yejee Lim, Jung Hun Ohn, Eun Sun Kim, Jiwon Ryu, Jangsun Kim, Yiseob Kim, Sun-wook Kim, Kwang-Il Kim

**Affiliations:** 1 Department of Internal Medicine Seoul National University Bundang Hospital Gyeonggi-do Republic of Korea; 2 Panoptics Corp Gyeonggi-do Republic of Korea

**Keywords:** smart diaper, urinary incontinence, enuresis, voided volume, diaper rash, smartphone, mobile phone, app, eHealth, mHealth, urine, medical device, sensor, prospective, pilot study, observational

## Abstract

**Background:**

Caregivers of patients who wear conventional diapers are required to check for voiding every hour because prolonged wearing of wet diapers causes health problems including diaper dermatitis and urinary tract infections. However, frequent checking is labor intensive and disturbs patients’ and caregivers’ sleep. Furthermore, assessing patients’ urine output with diapers in an acute care setting is difficult. Recently, a smart diaper system with wetness detection technology was developed to solve these issues.

**Objective:**

We aimed to evaluate the applicability of the smart diaper system for urinary detection, its accuracy in measuring voiding volume, and its effect on incontinence-associated dermatitis (IAD) occurrence in an acute care hospital.

**Methods:**

This prospective, observational, single-arm pilot study was conducted at a single tertiary hospital. We recruited 35 participants aged ≥50 years who were wearing diapers due to incontinence between August and November 2020. When the smart diaper becomes wet, the smart diaper system notifies the caregiver to change the diaper and measures voiding volume automatically. Caregivers were instructed to record the weight of wet diapers on frequency volume charts (FVCs). We determined the voiding detection rate of the smart diaper system and compared the urine volume as automatically calculated by the smart diaper system with the volume recorded on FVCs. Agreement between the two measurements was estimated using a Bland-Altman plot. We also checked for the occurrence or aggravation of IAD and bed sores.

**Results:**

A total of 30 participants completed the protocol and 390 episodes of urination were recorded. There were 108 records (27.7%) on both the FVCs and the smart diaper system, 258 (66.2%) on the FVCs alone, 18 (4.6%) on the smart diaper system alone, and 6 (1.5%) on the FVCs with sensing device lost. The detection rate of the smart diaper system was 32.8% (126/384). When analyzing records concurrently listed in both the FVCs and the smart diaper system, linear regression showed a strong correlation between the two measurements (*R^2^*=0.88, *P*<.001). The Bland-Altman assessment showed good agreement between the two measurements, with a mean difference of –4.2 mL and 95% limits of agreement of –96.7 mL and 88.3 mL. New occurrence and aggravation of IAD and bed sores were not observed. Bed sores improved in one participant.

**Conclusions:**

The smart diaper system showed acceptable accuracy for measuring urine volume and it could replace conventional FVCs in acute setting hospitals. Furthermore, the smart diaper system has the potential advantage of preventing IAD development and bed sore worsening. However, the detection rate of the smart diaper system was lower than expected. Detection rate polarization among participants was observed, and improvements in the user interface and convenience are needed for older individuals who are unfamiliar with the smart diaper system.

## Introduction

### Background

Urinary incontinence (UI), defined as the involuntary or abnormal loss of urine, represents a major health care problem in older adults [1]. UI is associated with falls and fractures, sleep disorders, and urinary tract infections. In addition, it decreases activity of daily living (ADL) and confidence and increases immobilization and hospitalization [2]. The prevalence of UI in a community population ranges from 5% to 45% [1,3-5] and steadily increases with age [5,6]. Furthermore, more than half of nursing home residents have incontinence [7-9], which is almost twice as high as the prevalence of community-dwelling individuals. Various treatments for UI, such as behavioral intervention, medication, and surgery, are limited in efficacy and may pose a challenge for frail geriatric patients. As a consequence, for older adults who are unable to take care of themselves, the most feasible options are wearing absorbent products or indwelling urinary catheters [9,10]. A previous study showed that more than 85% and 77% of older patients who are in long-term care facilities prefer using diapers and urinary catheterization, respectively [11].

However, wearing a diaper poses a number of risk factors. Incontinence care practices, such as prescheduled routine diaper checks, particularly at night, could be uncomfortable for the patients and disturb their daily living or sleep [12]. Wearing a wet diaper for a long time can lead to other health issues including diaper dermatitis (known as incontinence-associated dermatitis [IAD]) and bacterial infection [13,14]. Furthermore, IAD and infection cause or worsen bed sores, especially in bedridden older patients [13]. Indwelling urinary catheters are an alternative for UI, are not associated with IAD, and allow for accurate measurement of urine output in the acute care setting. However, it can cause urinary tract infections, and prolonged catheterization is associated with immobilization, loss of ADL, longer hospital stays, and mortality [15-17]. The best approach to prevent these complications is to replace unnecessary urinary catheterization with diaper use and to both detect the soiled diaper and change it as soon as possible. However, changing absorbent products in a timely manner is labor intensive for caregivers and health care providers, as most older patients with diapers have dementia or cognitive impairment. Moreover, recognizing urination without patient notification is difficult. Regularly scheduled diaper checks are not effective because the incontinence episodes of patients do not occur at precise times. In acute care hospital settings, measuring the weight of a wet diaper to assess input/output volume status increases the burden on caregivers. The onerousness of changing diapers may lead to unnecessary urinary catheterization and adversely affect patients’ morbidity and mortality.

Recently, the rapid development of Internet of Things (IoT) technology has changed intelligent health care systems. An increasing number of traditional health care services are being complemented or replaced with IoT [18]. Wireless technologies and miniaturized wearable sensing devices have contributed to several studies of smart diaper systems for incontinence care that detect moisture in absorbent products, inform caregivers to promptly change the wet diaper, and assess urinary output simultaneously [19-21]. Theoretically, the smart diaper system prevents the development of IAD or worsening of bed sores and provides a solution for urine output measurement without catheterization. Currently, some smart devices are being developed experimentally, but only a few clinical studies have been conducted on smart diapers. This study is the first to investigate the feasibility of a smart diaper system using Bluetooth and a smartphone to automatically detect urination and volume of voiding in an acute care hospital.

### Objectives

In this study, we aimed to evaluate the feasibility and utility of the smart diaper as an alternative to the conventional diaper in a clinical setting. We determined the detection rate of participants’ urination, accuracy of the urine output assessment, and occurrence of IAD. We also investigated the caregivers’ and health care providers’ experience and solicited suggestions.

## Methods

### Study Design and Population

This prospective, observational, single-arm pilot study was conducted at Seoul National University Bundang Hospital, a 1300-bed teaching hospital in Korea. From August to November 2020, participants were prospectively recruited at the hospitalist-run acute care unit and the geriatric center. Inclusion criteria were as follows: (1) patients aged ≥50 years, and (2) those who wore diapers due to incontinence. Participants who wore diapers temporarily at night or for part of the day, rather than 24 hours per day, were excluded. Demographic data, laboratory data, and previous medical history were retrieved from electronic medical records.

The participants received diaper pads for three days. They could extend the participation period up to two days, for a total of five days, if they wanted to continue using smart diapers on the third day of the study and had to remain hospitalized for medical reasons. The caregiver of each participant was provided with sets of the smart diaper system and trained daily by a researcher on how to use them. In response to the smart diaper system’s alarm, caregivers checked voiding, changed the diaper, weighed the wet diaper on a digital scale, and recorded the weight of the wet diaper on the frequency volume charts (FVCs). The researcher subtracted the weight of an unused diaper (110 g) from the weight of the wet diaper as recorded on the FVCs; this is considered the gold standard for determining urine volume. The manually measured urine output was compared with the urine output automatically detected by the smart diaper system to determine the accuracy of the smart diaper system. In addition, the researcher visited the participants once daily to check for the development or aggravation of IAD and record any evidence of IAD by taking photographs. Color, location, size, and character (scale, crust, discharge, etc) of IAD, as well as the presence of bed sores, were documented. Two investigators reviewed the participants’ skin, and another investigator was consulted if any disagreement was found between the first two investigators. At the end of the trial, each caregiver completed a questionnaire on the user experience of the smart diaper system.

### Materials

**Figure 1:**
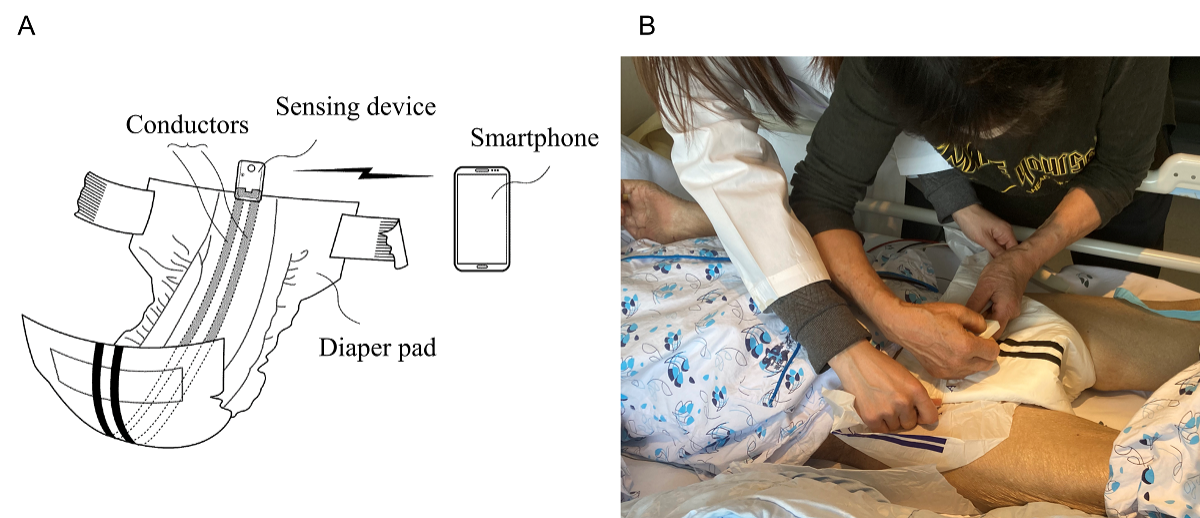
The smart diaper system. (A) The design of the system consists of a diaper embedded with two lines of conductors, a sensing device, and a smartphone. (B) A caregiver connects the sensing device to a smart diaper under a researcher’s guidance.

The smart diaper system consists of three parts: (1) a diaper embedded with conductors, (2) a sensing device, and (3) a smartphone (Figure 1). The inner surface of the diaper contains an absorbent liner for urine, and the outer surface has two lines of embedded conductors, which are printed at 1-cm intervals using carbon paste conductive ink. The two lines of conductors with regular spaces are connected to the sensing device at the front end of the diaper. The sensing device induces a minute electrical current, which a human cannot perceive, to the first conductor at regular time intervals. The current that passes through the second conductor is detected by the sensing device. When the diaper is wet, the current flow between the first and second conductors increases. As the amount of urine increased, the increment in amplitude of induced voltage increased. Therefore, the sensing device could detect subject’s voiding and measure the amount of urine produced. The smartphone received a regular signal from the sensing device via Bluetooth. If the amount of urine exceeded a pre-established threshold, a dedicated app installed on the smartphone informed the caregiver about the patient’s urination records. In this study, the minimum and maximum values of detectable urine volume were set to 50 mL and 500 mL, respectively. The diaper embedded with conductors obtained product certification as a personal hygiene item from the Korea Apparel Testing and Research Institute. There is a Korean patent pending for the whole system (10-2020-0093675).

### Ethics Statement

All procedures performed in this study involving human participants were in accordance with the 1964 Declaration of Helsinki and its later amendments or comparable ethical standards. The study protocol was reviewed and approved by the Institutional Review Board of Seoul National University Bundang Hospital (B-2007/627-307). A researcher explained the study to the participants and their legal representatives and obtained written informed consent.

### Statistical Analysis

Demographic data and baseline characteristics of the participants were analyzed using descriptive statistics. Data are presented as mean (SD) for continuous variables or counts with percentages for categorical variables. Linear regression analysis was used to evaluate the association between the manually weighed urine volume and the urine volume calculated by the smart diaper system. The correlation was described by the *R^2^* and plotted in a scatter plot. The agreement between the two measurements was analyzed by the Bland-Altman method [22,23]. The mean difference (bias) and upper and lower limits of agreement, defined as the mean difference ± 1.96 SD of differences, were calculated and constructed graphically with a Bland-Altman plot. A *P* value of <.05 was considered statistically significant, and all analyses were two-tailed. We logged and analyzed the data using SPSS (version 25.0; IBM Corp) and MedCalc (MedCalc Software). All data from the participants were deidentified and analyzed anonymously.

Calculation of a formal sample size was not performed in this study because this was a pilot study and estimable data from previous studies were unknown. However, a general rule of thumb is to include ≥30 patients to estimate a parameter [24]. Therefore, we recruited 35 patients, assuming approximately a 15% dropout rate.

## Results

### Baseline Characteristics

**Figure 2:**
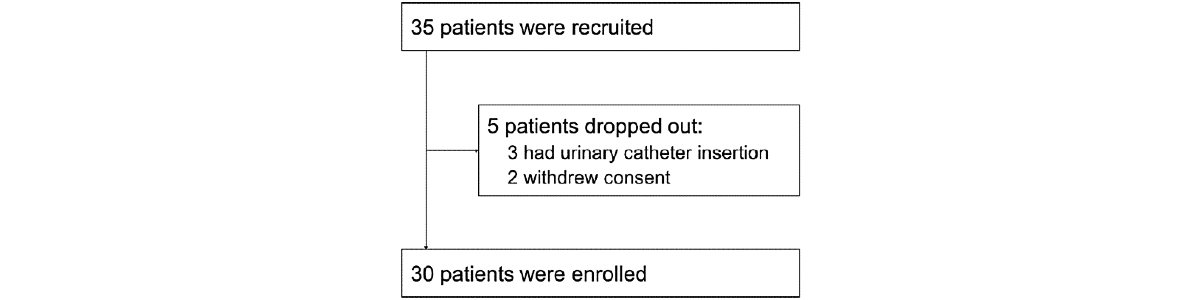
Flow diagram of study participants.

Of 35 participants, urinary catheterization was performed in three and two withdrew consent before data collection. Accordingly, data from 30 participants was used for the analysis (Figure 2). The demographic characteristics of the participants are described in Table 1. The mean age was 80.8 years, and 9 (30%) participants were men. Table 1 shows the commonly recorded diagnoses, including hypertension (23/30, 77%), diabetes (17/30, 57%), dementia (6/30, 20%), and Parkinsonism (2/30, 7%); in addition, 10 participants (33%) had a history of stroke. Serum total cholesterol, total protein, and albumin levels were lower than the normal range, suggesting risk of malnutrition.

**Table 1:** Baseline characteristics of the study population.

Variables			Population				
			All (N=30)		Male (n=9)		Female (n=21)
**Demographic data, mean (SD)**							
	Age (years)	80.8 (7.6)		82.1 (7.9)		80.2 (7.6)	
	Height (cm)	157.0 (7.8)		164.3 (4.7)		153.9 (6.8)	
	Weight (kg)	50.4 (8.1)		53.1 (7.9)		49.2 (8.0)	
	BMI (kg/m^2^)	20.4 (2.9)		19.7 (3.1)		20.8 (2.9)	
**Laboratory data, mean (SD)**							
	White blood cell (×10^3^/μL)	9.0 (4.0)		10.1 (4.4)		8.6 (3.9)	
	Hemoglobin (g/dL)	9.9 (1.7)		10.1 (2.1)		9.8 (1.6)	
	Platelet (×10^3^/μL)	188.9 (93.7)		171.1 (111.8)		196.6 (86.8)	
	Blood urea nitrogen (mg/dL)	20.0 (9.6)		18.1 (7.2)		20.8 (10.5)	
	Creatinine (mg/dL)	0.9 (0.5)		1.0 (0.5)		0.9 (0.5)	
	Total cholesterol (mg/dL)	119.1 (38.7)		110 (48.6)		123.0 (34.2)	
	Protein (g/dL)	5.9 (1.0)		6.0 (1.3)		5.9 (0.8)	
	Albumin (g/dL)	2.9 (0.6)		2.7 (0.7)		3.0 (0.6)	
**Previous medical history, n (%)**							
	Hypertension	23 (77)		6 (67)		17 (81)	
	Diabetes	17 (57)		6 (67)		11 (52)	
	History of stroke	10 (33)		3 (33)		7 (33)	
	Dementia	6 (20)		2 (22)		4 (19)	
	Parkinsonism	2 (7)		1 (11)		1 (5)	
	Malignancy	11 (37)		4 (44)		7 (33)	

### Characteristics of Urination Records

**Figure 3:**
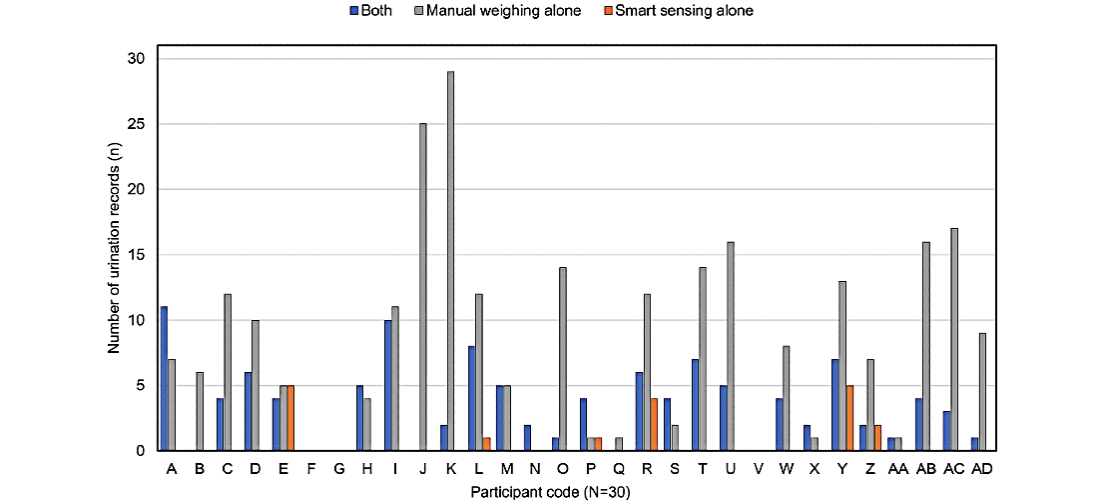
Histogram of distribution of urination records for each participant. There were no records available for 3 participants (F, G, and V).

In total, 401 urination records found on the FVCs or the smart diaper system were collected, and 11 records reported 50 mL or less of urine. Since we had set the smart diaper system to not detect urine volumes less than 50 mL, we excluded those 11 records. Finally, 390 urination records were available for analysis. There was an average of 13.0 urination records per participant during the study period and an average of 3.7 urination records per person per day. The classification of 390 urination records is shown in Table 2. There were 108 paired records (27.7%) on the FVCs and the smart diaper system concurrently, 258 (66.2%) on the FVCs alone, 18 (4.6%) on the smart sensing system alone, and 6 (1.5%) on the FVCs with the sensing device lost so the smart diaper system could not detect urination. The detection rate of the smart diaper system was 32.8% (126/384), excluding 6 records where the sensing device was lost. The detailed urination records for each participant are shown in Figure 3. It should be noted that two participants (anonymous patient codes F and G) presented no record at all, and one participant (V) presented only urination records in which the urine volume measured by manual weighing was 50 mL or less.

**Table 2:** Classification of a total of 390 urination records collected from 30 participants^a^.

Variables			Value (N=390), n (%)
**Urination records, n (%)**			
	Both manual weighing and smart sensing records	108 (27.7)	
	Only manual weighing record	258 (66.2)	
	Only smart sensing record	18 (4.6)	
	Sensing device lost	6 (1.5)	

^a^No data was available from 3 participants.

### Correlation Between the Two Methods

**Figure 4:**
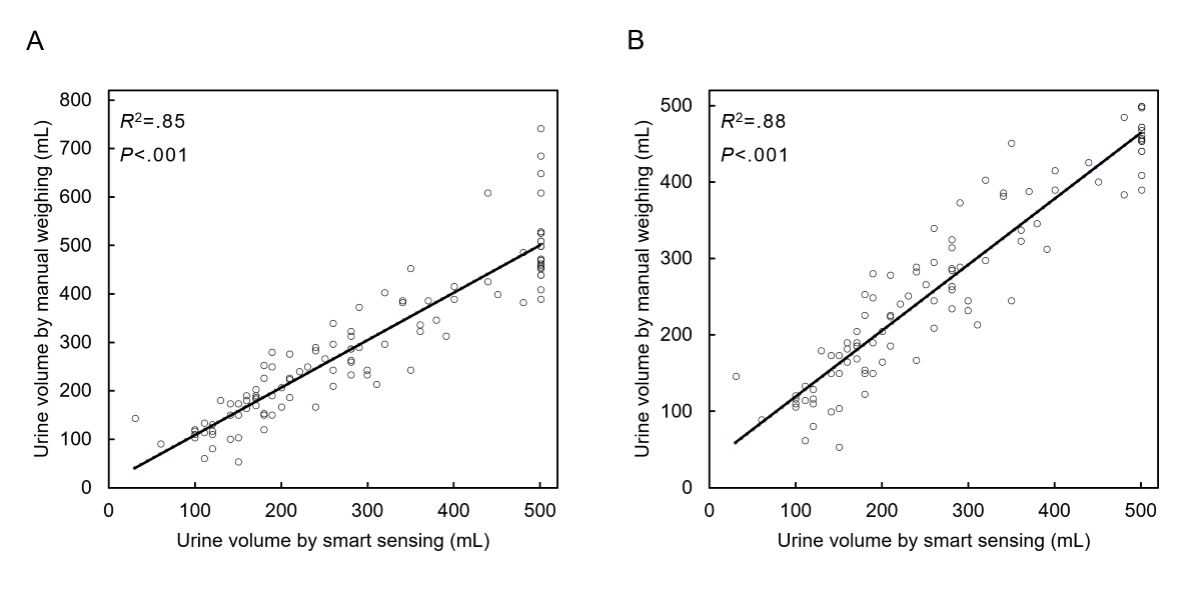
Scatter plot of urine volume measured by manual weighing and the smart diaper system. A significant linear regression relationship was found between the two measurements in the 108 urination records (A) and the 98 records (B).

To verify the accuracy in urine volume measurement of the smart diaper system, we evaluated the relationship and agreement between the two measurements: weighing wet diapers manually on a digital scale and automatic detection by smart sensing. In addition to the analysis of the 108 paired records, subgroup analysis was performed excluding 10 paired records containing a manually measured volume of voiding over 500 mL (ie, greater than the maximum detectable value for the smart diaper system). A linear regression showed a strong correlation between the two measurements for the 108 records (*R^2^*=0.85, *P*<.001) and the 98 subgroup records (*R^2^*=0.88, *P*<.001), respectively (Figure 4).

### Agreement Between the Two Methods

**Figure 5:**
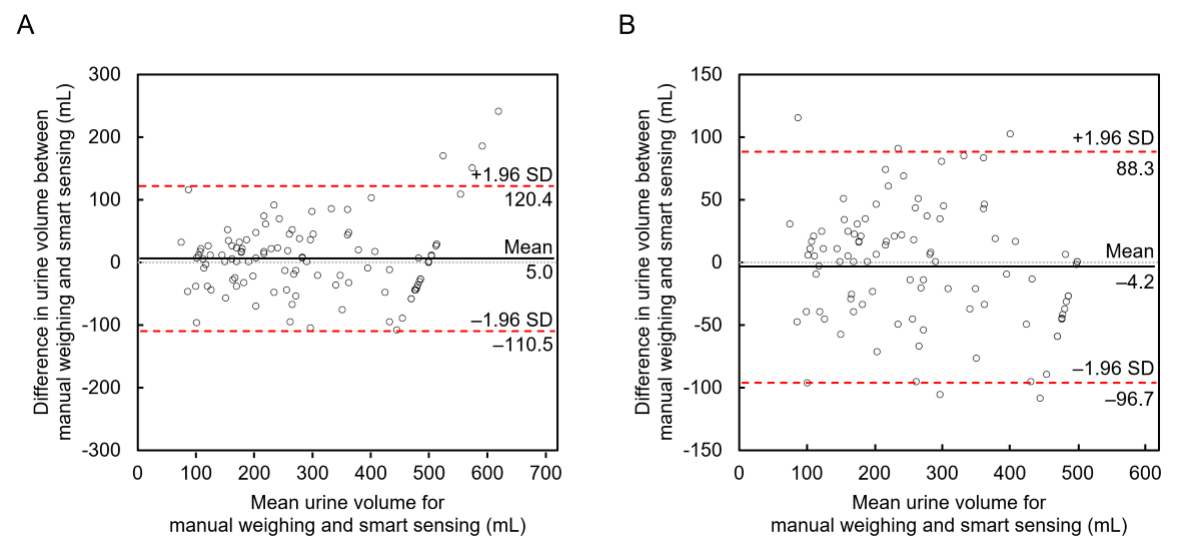
Bland-Altman plot of urine volume measured by manual weighing and the smart diaper system in the 108 urination records (A) and the 98 records (B). Mean (mean difference; solid line), 95% limits of agreement (dashed lines), and line of equality (dotted line) are shown.

Bland-Altman analysis was performed for agreement between the two measurement techniques (Figure 5). In the analysis of the 108 records, the mean difference between the two measurements was 5.0 mL, and the lower and upper limits of agreement were –110.5 mL and 120.4 mL, respectively. In the analysis of the 98 subgroup records, the mean difference between the two measurements was –4.2 mL, and the lower and upper limits of agreement were –96.7 mL and 88.3 mL, respectively. Based on the analysis of the 98 subgroup records, the 95% limits of agreement of all paired data were deemed clinically interchangeable. Meanwhile, the average (SD) of the absolute values (|x|) of the difference between the two measurements was 42.6 mL (SD 40.9 mL) for the 108 records and 37.2 mL (SD 29.0 mL) for the 98 subgroup records, respectively.

### IAD, Bed Sores, and Adverse Events

A total of 28 participants were evaluated for IAD and analyzed for bed sores (2/30 enrolled participants refused photography). Prior to the start of the study, 8 participants already had bed sores: 6 with grade 2 (partial loss of skin layers) and 2 with grade 3 (necrosis of subcutaneous tissue). At the end of the study, the sores had not increased in size or degree. An improvement from bed sore grade 3 to 2 was noted in one participant. In the remaining 20 participants, de novo IAD or bed sores were not observed. Moreover, during the study period, no adverse events were reported.

### User Experience

After the study, caregivers answered a series of survey questions. A total of 22 caregivers completed the questionnaire. The respondents consisted of 12 (54%) employed caregivers, 9 (41%) offspring, and 1 (4%) sibling. The respondents were asked about their willingness to purchase the smart diaper system once it becomes commercialized. About 59% (13/22) and 41% (9/22) had positive and negative responses, respectively. They were also asked about inconveniences related to using the smart diaper system. Cumbersome connections between the diaper and the sensing device or Bluetooth pairing between the sensing device and the smart phone were reported by 6 (27%) of the respondents, and intermittent breakdown of the smart diaper system was reported by 9 (41%; Multimedia Appendix 1).

## Discussion

### Principal Findings

The objectives of this study were to evaluate the feasibility and utility of the smart diaper system as an alternative to the conventional diaper in a clinical setting. We found that the accuracy of automated urine volume measurement by the smart diaper system compared to the conventional wet diaper weighing method was clinically acceptable. We also found that the smart diaper system had the potential advantage of preventing IAD development and the worsening of bed sores. However, the detection rate of the smart diaper system was low. A large variation in detection rate was observed among participants. Furthermore, we found the inconvenience of the operation and user interface of the smart diaper system presented a problem.

### Smart Diaper System in Real Life

This study demonstrated that a smart diaper system developed with IoT technology is applicable in real life, including hospital settings. In acute care hospitals, measuring urine output at regular intervals is often necessary to control the patient’s body fluid and electrolyte homeostasis. Therefore, for patients with diapers, health care providers should determine urine volume by counting the number of diaper changes or weighing wet diapers at each change. However, inference based on the number of diapers used is inaccurate and there is often poor compliance for measuring wet diapers. Urinary catheterization is another alternative for more accurate measurement of urine volume, although it is associated with increased urinary tract infections and morbidity. In this study, the average of the absolute values of the difference between urine volume measured by weighing wet diapers on a digital scale and urine volume measured by the smart diaper system was 42.6 mL, which was 14.3% of the average urine volume per urination of 297.2 mL in the 108 paired urination records. Excluding 10 urination records of >500 mL, which was the upper limit of detection of the smart diaper system, the level of difference between the two measurements was only 13.9% (37.2 mL/267.4 mL in the 98 urination records), suggesting clinical acceptability. Consequently, our study suggested that the urine volume measurements obtained using the smart diaper system were more reliable than estimation by counting the number of urinations, more convenient than weighing wet diapers, and less invasive than an indwelling urinary catheter.

### Consideration for Low Detection Rate

In this study, the overall voiding detection rate of the smart diaper system was 32.8% (126/384), which was lower than expected. As previously mentioned, considering the small margin of error in urine volume measurement between the two techniques, it was a questionable finding. The detection rate varied widely from participant to participant (range 0%-100%). Figure 3 shows that a large number of voiding detections by the smart diaper system were from a subset of participants, but in 13 participants, less than three counts of voiding detection by the smart diaper system were recorded during the study period. Consequently, we can infer two reasons for this finding. First, when water was poured on the smart diaper pad during the development process, there was a delay of 1-20 minutes in detecting wetness due to the preset time interval of sensing and the Bluetooth connection to save battery power. If the participant had immediately informed the caregiver of urination themselves, or if the caregiver had recognized the participant's urination promptly, diapers could have been changed before detection by the smart diaper system. Second, the caregivers may have had poor understanding and compliance regarding using the smart diaper system appropriately. For the smart diaper system to work well, a good connection between the diaper pad and the sensing device is crucial. Bluetooth pairing between the smartphone and the sensing device is also required. Although a researcher visited and trained the caregiver daily, skillful handling of the first smart diaper system might have been difficult for caregivers. The smart diaper system works well with the researchers’ assistance; however, it could be challenge for older caregivers to learn to use the system within the short study period of three days.

### Consideration for IAD and Bed Sores

According to the literature, IAD incidence has been reported to be 36% in critically ill patients [25], 7.6% in long-term acute care patients [26], and 5.5% in nursing home residents with new-onset incontinence [27]. IAD may increase the risk of new bed sores, be more frequent in patients with bed sores, and even worsen existing bed sores [28]. In our study, no newly developed dermatitis or bed sores were detected in participants. In addition, aggravation of bed sores was not observed in participants who previously had bed sores, and one case of improvement of bed sores was observed. Timely changing of wet diapers and keeping the area near the perineum dry using the smart diaper system contributed to this effect.

### Aged Society and Digital Medicine

As the population ages, the health care expenditures for older adults with multimorbidity are expected to increase steeply. Moreover, increasing socioeconomic gaps can lead to disparities in the health and wellbeing of older people. Digital medicine is an emerging solution for effective control of chronic medical conditions in geriatric populations at a relatively low cost [29]. For the last two decades, eHealth has revolutionized the medical sector with computers, the internet, and electronical medical record systems [30,31]. More recently, the advent of IoT technology and the widespread use of mobile devices have led to the development of mobile health (mHealth), a subdivision of eHealth, which has the potential to improve health outcomes in chronic disease management [32,33]. However, for now, many older adults and their caregivers have low digital literacy when it comes to using the internet, smartphones, and IoT technology [34]. Therefore, more research about geriatric digital medicine and further development of age-friendly user interfaces are urgently needed to ensure older adults can benefit from digital health.

### COVID-19 Pandemic and Digital Medicine

Since the SARS-CoV-2 outbreak started in December 2019, there have been unprecedented impacts across the world. In the era of COVID-19, social and economic activities are changing to non–face-to-face platforms, and health care systems are facing significant challenges related to converting to and using telemedicine. Virtual connections between tertiary hospitals and long-term care facilities were activated in many countries, and “hospital at home,” which provides hospital-level care in a patient’s home as a substitute for acute hospital care, is emerging as another option in the post–COVID-19 era [35]. In these “contact-free” health care environments, the demand for digital medicine including mHealth is also increasing, and the smart diaper system is expected to play an important role. The smart diaper system could monitor voiding and urine volume, analyze daily or weekly patterns, and transfer the data to medical professionals to help patients at home or in long-term care facilities.

### Limitations and Strength

This study had several limitations. First, the study population was small. Second, the study period was only a few days, which might not have provided the caregivers with sufficient time to adapt to the digital equipment. In addition, the study duration may not have been sufficient to observe skin changes in the diaper area, such as IAD and bed sores. Nonetheless, it was a pilot study, and the applicability of the smart diaper system was established. Further large-scale and long-term investigations with age-friendly equipment and user interface are warranted for commercialization and popularization of the smart diaper system. Third, some participants were less compliant, and they did not report any voiding record. A survey conducted after the study showed that 27% (6/22) of the respondents reported inconvenience in using the smart diaper system and 41% (9/22) reported difficulties in operating the smart diaper system. Therefore, developing a more user-friendly system is necessary to improve compliance. Fourth, we did not perform a qualitative and quantitative usability assessment using validated questionnaires, although we reviewed the user experience through a survey after the study.

Nonetheless, the strength of this study is that it provides data on experiences using the smart diaper system in a clinical setting. To the best of our knowledge, this study is the first to investigate the feasibility of a smart diaper system in an acute care hospital. The only previous study was conducted with 18 people with dementia living in nursing homes and estimated the saturation of diaper capacity indirectly rather than measuring the volume of urine [36]. The study reported that the saturation errors between the smart diaper system and FVCs were –26% to 39% in the regular diaper (450 mL) with 51 urination records, and –34% to 30% in the super diaper (1000 mL) with 46 records, respectively. The study did not provide information about the detection rate of the system and concluded that it was not sensitive enough to use as an indicator of the need for a diaper change.

### Conclusions

This study suggests that the smart diaper system can promptly notify caregivers of patients’ urination to facilitate diaper changes and prevent the occurrence of IAD and bed sores or improve existing IAD and bed sores by reducing exposure time to wet diapers. In addition, the smart diaper system can measure urine volume with reliable accuracy and less invasiveness than the conventional weighing method, reducing the need for routine diaper checks. Therefore, it can save the time and labor of caregivers, as well as reduce patient discomfort. The smart diaper system is expected to help many health care facilities and people in need of care.

## Appendix

Multimedia Appendix 1 Results from a questionnaire on the user experience of the smart diaper system.

## Abbreviations

ADL: activity of daily living
FVC: frequency volume chart
IAD: incontinence-associated dermatitis
IoT: Internet of Things
mHealth: mobile health
UI: urinary incontinence
