# Supplementary material for: A Smart Diaper System Using Bluetooth and Smartphones to Automatically Detect Urination and Volume of Voiding: Prospective Observational Pilot Study in an Acute Care Hospital
Source: J Med Internet Res. 2021 Jul 30;23(7):e29979. doi: 10.2196/29979 (PMC8367151; doi:10.2196/29979)
Supplement: Multimedia Appendix 1 [file jmir_v23i7e29979_app1.docx]

| Table S1. Result of a questionnaire on the user experience of the smart diaper system. | | | |
| --- | --- | --- | --- |
| Questions | | | Value  (N=22) |
| **Would you like to buy and use the smart diaper system if it is going to be released on the market?** | | | |
|  | I am willing to purchase with priority regardless of the price. | | 3 (13.6%) |
|  | I am willing to purchase at a reasonable price. | | 10 (45.5%) |
|  | No. I am going to use the conventional diapers. | | 9 (40.9%) |
| **What discomfort have you experienced while using the smart diaper system?** | | | |
|  | **A. About device connections** | | |
|  |  | Connecting the sensing device to the diaper. | 3 (13.6%) |
|  |  | Bluetooth paring between the sensing device and the smart phone. | 3 (13.6%) |
|  | **B. About the alarm** | | |
|  |  | The alarm did not work, when voiding. | 7 (31.8%) |
|  |  | The alarm worked without voiding. | 2 (9.1%) |

**Multimedia Appendix 1. Supplementary table.**
